# Insights into SARS-CoV-2: Medicinal Chemistry Approaches to Combat Its Structural and Functional Biology

**DOI:** 10.1007/s41061-021-00335-9

**Published:** 2021-04-22

**Authors:** Lin-Sheng Zhuo, Ming-Shu Wang, Jing-Fang Yang, Hong-Chuang Xu, Wei Huang, Lu-Qing Shang, Guang-Fu Yang

**Affiliations:** 1 Key Laboratory of Pesticide and Chemical Biology of Ministry of Education, International Joint Research Center for Intelligent Biosensor Technology and Health, College of Chemistry, Central China Normal University, Wuhan, 430079 People’s Republic of China; 2 College of Pharmacy, State Key Laboratory of Medicinal Chemical Biology and Tianjin Key Laboratory of Molecular Drug Research, Nankai University, Tianjin, 300350 People’s Republic of China

**Keywords:** SARS-CoV-2, COVID-19, Structural biology, Antiviral, Drug discovery

## Abstract

Coronavirus disease 2019, caused by the severe acute respiratory syndrome coronavirus 2 (SARS-CoV-2), is still a pandemic around the world. Currently, specific antiviral drugs to control the epidemic remain deficient. Understanding the details of SARS-CoV-2 structural biology is extremely important for development of antiviral agents that will enable regulation of its life cycle. This review focuses on the structural biology and medicinal chemistry of various key proteins (Spike, ACE2, TMPRSS2, RdRp and M^pro^) in the life cycle of SARS-CoV-2, as well as their inhibitors/drug candidates. Representative broad-spectrum antiviral drugs, especially those against the homologous virus SARS-CoV, are summarized with the expectation they will drive the development of effective, broad-spectrum inhibitors against coronaviruses. We are hopeful that this review will be a useful aid for discovery of novel, potent anti-SARS-CoV-2 drugs with excellent therapeutic results in the near future.

## Introduction

COVID-19, caused by the novel coronavirus SARS-CoV-2, has developed into a serious public health event around the world. To date, more than 108 million individuals have been infected by SARS-CoV-2 in at least 210 countries/territories [1]. Like MERS-CoV and SARS-CoV, SARS-CoV-2 belongs to the beta-coronavirus family that is thought to spread mainly through person-to-person contact and air. Their positive, single-strand RNA genomes are a typical family characteristic, exhibiting a spherical protein envelope and diameter of about 65–125 nm [2].

**Fig. 1:**
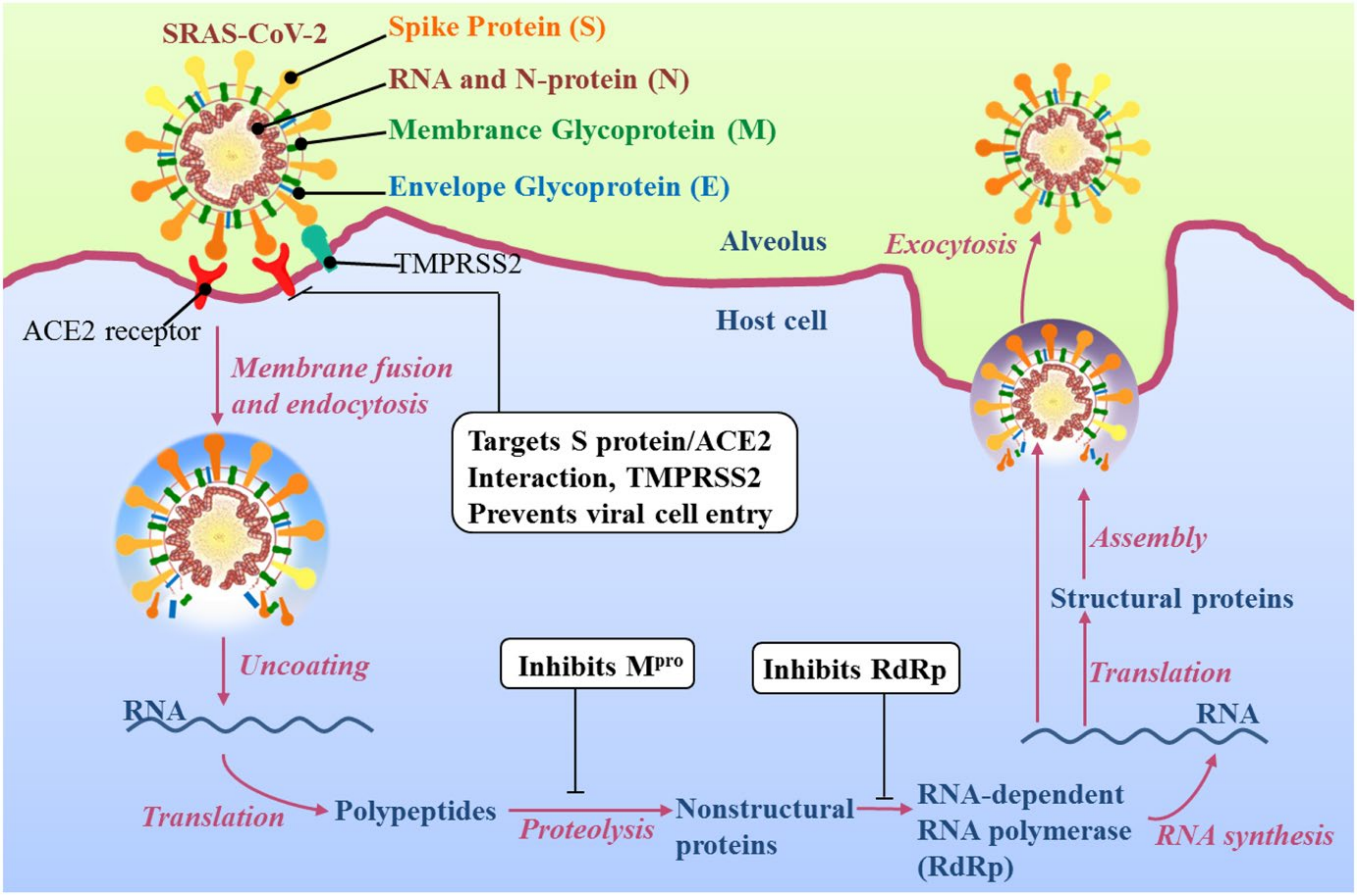
The life cycle of SARS-CoV-2, including: membrane fusion, endocytosis, uncoating, translation of RNA, proteolysis, assembly and exocytosis

Liu and co-workers reported an electron microscopy image of isolated SARS-CoV-2 [3]. The envelope consists of Spike protein (S), RNA and nucleocapsid-protein (N), membrane glycoprotein (M) and envelope glycoprotein (E) (Fig. 1). The S protein (also named S glycoprotein), embedded in the virus envelope, is a transmembrane protein with molecular weight of about 150 kDa. In the process of cell entry (Fig. 1), the S protein acts in a pivotal role during invasion of SARS-CoV-2 into host cells. It enters via interaction with angiotensin-converting enzyme 2 (ACE2) receptors located on the cell surface of type II pneumocytes [4]. Enzymatic hydrolysis of the complex between SARS-CoV-2 and host cells is processed by the type II transmembrane serine protease TMPRSS2. After cleavage of ACE-2 and activation of S protein, SARS-CoV-2 fuses to the host cell membrane [5]. Blocking the S protein/ACE2 interaction, or deactivating TMPRSS2, have been identified as promising strategies to prevent viral cell entry. However, once fusion occurs after uncoating of the SARS-CoV-2 envelope, the positive single-strand RNA is released in the cytoplasm and moves to the cell nucleus whereupon it becomes translated to produce nonstructural proteins and viral polymerases. After advancing through the sequence of translation-proteolysis-RNA synthesis, viral structural proteins are synthesized from subgenomic mRNA, and these are further packaged into new virion assemblies [6]. Importantly, a variety of nonstructural proteins exhibit multiple functions that affect the survival and toxicity of coronaviruses. For example, the main protease (M^pro^) promotes cytokine expression and cleavage of viral polyproteins. The RNA-dependent RNA polymerase (RdRp) takes a central role in the replication of coronavirus (Fig. 1) [7, 8]. As M^pro^ and RdRp act a pivotal part in replication and transcription of coronavirus, extensive effort has been devoted to the development of M^pro^ and RdRp inhibitors to control diseases caused by coronavirus.

In light of the infectious route outlined above, and with a view to uncover new inhibitors/drug candidates that interrupt this paradigm, our perspective focuses on understanding the structural biology and medicinal chemistry of various important proteins (Spike, ACE2, TMPRSS2, RdRp and M^pro^) in the life cycle of SARS-CoV-2. The representative broad-spectrum antiviral drugs, especially those against homologous virus SARS-CoV, are also summarized with the expectation that may aid in driving the development of anti-SARS-CoV-2 drugs.

## S protein, ACE2 and TMPRSS2, the Key Proteins for Cell Entry of SARS-CoV-2

Numerous studies have demonstrated that virus infection begins with the recognition and binding of the virus to the receptors on the host cell surface [9]. For coronaviruses, entry into host cells occurs through receptor-mediated recognition by the transmembrane S protein followed by membrane fusion [4, 10, 11]. Since SARS emerged in 2002, the structural biology-based mechanisms of infection by SARS-CoV have been widely studied. The human angiotensin I-converting enzyme 2 (*h*ACE2) was proven to be the main entry receptor. Once it is recognized by the SARS-CoV S protein, the cellular serine protease TMPRSS2 is recruited to cleave the viral S protein, an obligatory action that must occur before membrane fusion [4, 12–14]. Recent studies indicate that SARS-CoV-2 also takes advantage of the host proteins TMPRSS2 and ACE2 for cell entry. Recent evidence shows that the affinity of SARS-CoV-2 S protein to ACE2 is higher than that of SARS-CoV. This fact may be the reason for higher rates of transmission of SARS-CoV-2 in humans [15].

### The Structure of SARS-CoV-2 S Protein

**Fig. 2:**
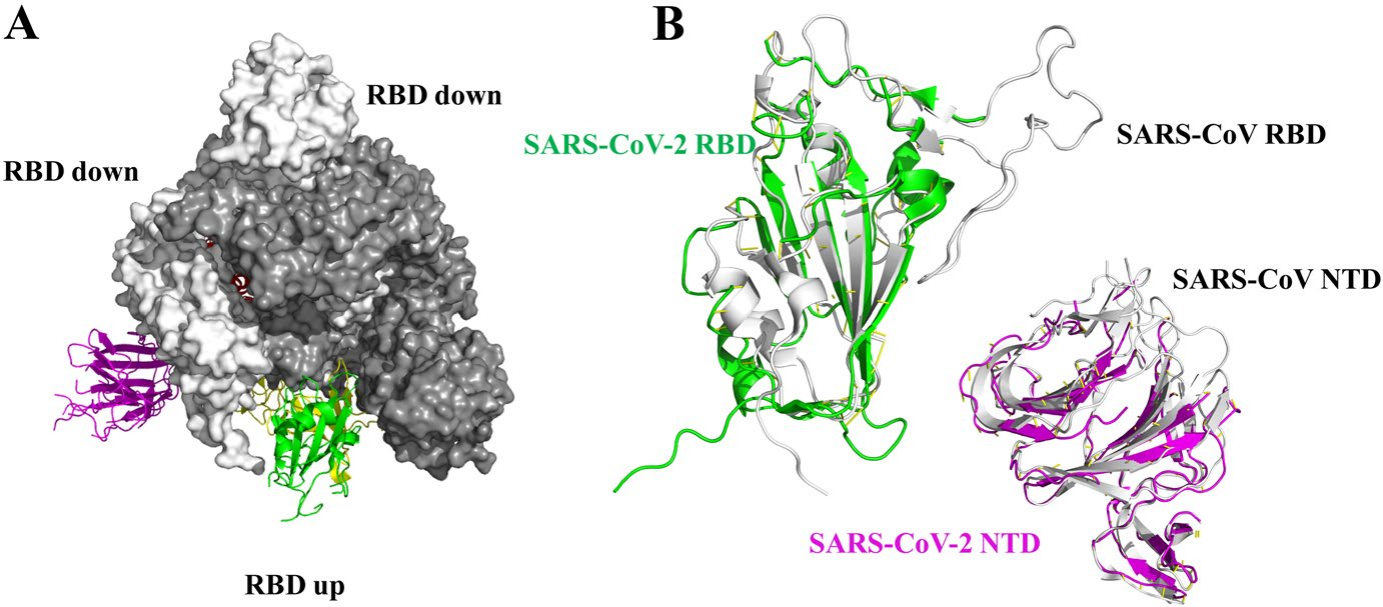
**a** Cryo-EM structure of the SARS-CoV-2 S protein in the prefusion conformation (PDB ID 6VSB) [15]; **b** an overlay of RBDs from SARS-CoV-2 and SARS-CoV based on the position of their respective adjacent NTD: SARS-CoV-2 RBD (green), SARS-CoV RBD (white), SARS-CoV-2 NTD (purple), SARS-CoV NTD (white) [15]

The S protein is a trimeric, class I fusion protein with homotrimers protruding from the viral surface and two functional subunits located near the amino (S1) and carboxy (S2) termini [15–17]. The virus uses the S1 subunit for binding to the receptors of the host cell. The S2 subunit is employed for the virus-host membrane fusion [18]. Like other class I fusion glycoproteins, membrane fusion activity depends on the proteolytic cleavage of S protein into S1 and S2 subunits. Cell entry of SARS-CoV-2 is accomplished by the engagement between a receptor-binding domain (RBD) in the homotrimeric S protein and ACE2 receptor followed by virus-host membrane fusion [19, 20]. Destroying the key role of S protein in the process of infection is the main purpose of neutralizing antibodies and the interest of vaccine design as well as therapeutic interventions [21]. Wrapp et al. first used cryo-electron microscopy (cryo-EM) to investigate the SARS-CoV-2 S protein, and reported the structure in the pre-fusion conformation (Fig. 2a) [15]. They obtained the crystal structure of an asymmetrical trimer at 3.5-Å resolution. As shown in Fig. 2a, the single RBD presents an up conformation that corresponds to the receptor-accessible state. They also demonstrated that the structures of different domains, including RBDs, N-terminal domains (NTDs), SD1/2s and S1/2s in SARS-CoV-2, have a high degree of homology with corresponding domains in SARS-CoV. The root mean square difference (RMSD) values range from 2.0 to 3.0 Å. [15]. As shown in Fig. 2b, the noteworthy difference between these two S proteins from SARS-CoV-2 and SARS-CoV is the position of the RBDs. The SARS-CoV RBD highlighted by the white color packs tightly against the NTD of the adjacent protomer, while the angle of SARS-CoV-2 RBD is closer to the core cavity of the trimer [15].

In the novel coronavirus of most recent interest, an “RRAR” furin recognition site was observed at the boundary near the S1/S2 protease cleavage site that is often found to be related to high virulence [4, 16, 22, 23]. Walls et al. examined cryo-EM structures of the SARS-CoV-2 S ecto-domain trimer, and made a detailed comparison with that of SARS-CoV [4]. They observed that the SARS-CoV-2 S protein exists in a cross-domain conformation that appears to be a 160-Å-long trimer, which closely resembles the structure of the S protein of SARS-CoV. Within the trimer, the S1 subunit presents a V-shaped structure, while the S1 subunit is in an extended conformation. Walls et al. also identified that in the SARS-CoV-2 RBD, a structural opening is essential to initiate a conformational change for recognition of ACE2 that then leads to membrane fusion and cell entry. They also found a 75% sequence homology in the RBD and 88% sequence homology in the S2 subunit between SARS-CoV and SARS-CoV-2. Overall, the structure to emerge from the SARS-CoV-2 S protein is similar to that of all reported coronaviruses, especially to that of SARS-CoV.

**Fig. 3:**
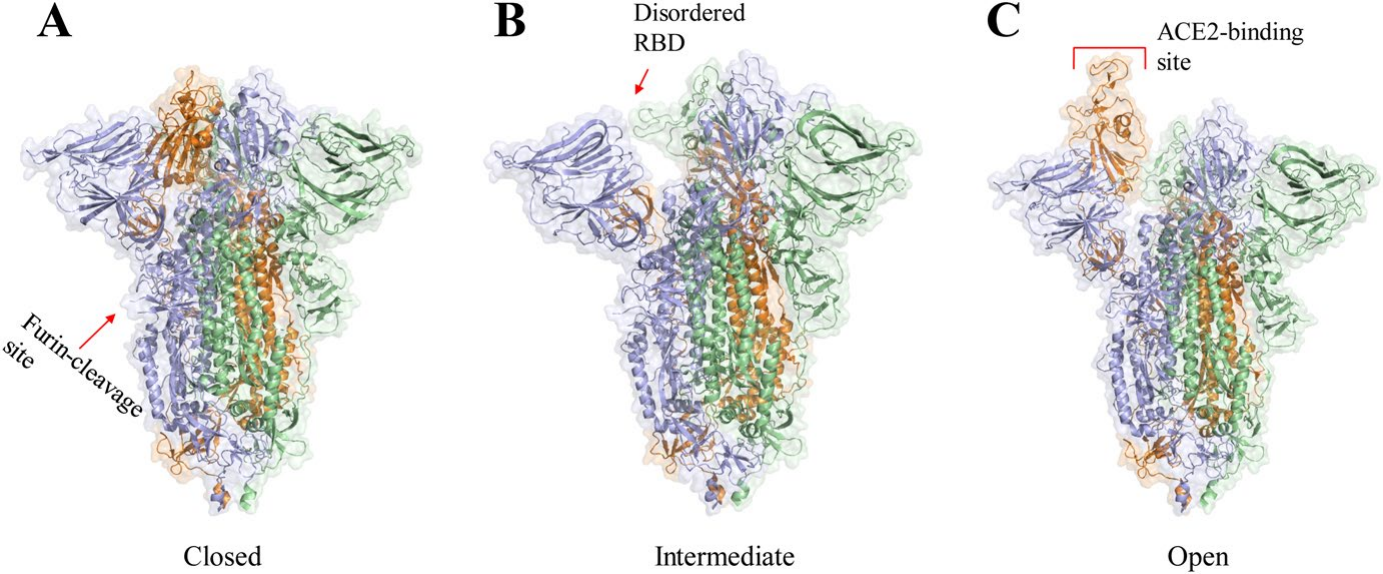
The three structures of S protein of SARS-CoV-2 are calculated from micrographs of furin-cleaved material: **a** closed (PDB ID 6ZGI); **b** intermediate (PDB ID 6ZGH); **c** open (PDB ID 6ZGG) forms. The three monomers are colored orange, light blue and pale green [24]

To investigate the evolutionary origin of SARS-CoV-2 and gain better insight into the emergence of the COVID-19 pandemic, Wrobel et al. [24] characterized the structures of S protein of SARS-CoV-2 virus (Fig. 3). In the closed conformation (Fig. 3a), the surface of the RBD is wrapped in the interior of the trimer, which makes it unable to interact with the ACE2 receptor. Furin cleavage sites associated with increased pathogenicity were observed between S1 and S2 subunits. In the intermediate conformation (Fig. 3b), one of the three RBDs is disordered, while the other two keep a similar conformation with the closed form. Moreover, the centroids of the two NTDs that are in closest contact with the disordered RBD are shifted by 2.5–2.9 Å compared to that of closed form. The disordered RBD in the intermediate form leads to a decrease in the stability of the S protein, which ultimately enables the formation of an open form. In the open conformation (Fig. 3c), one of the RBDs is completely exposed to the ACE2-interacting surface after rotating ~ 60°, and the other two RBDs are consistent with that of closed and intermediate forms. This rotation is facilitated by the movement of NTD in the adjacent and same chain.

### The Structural Basis of *h*ACE2 Recognizing S Protein of SARS-CoV-2

**Fig. 4:**
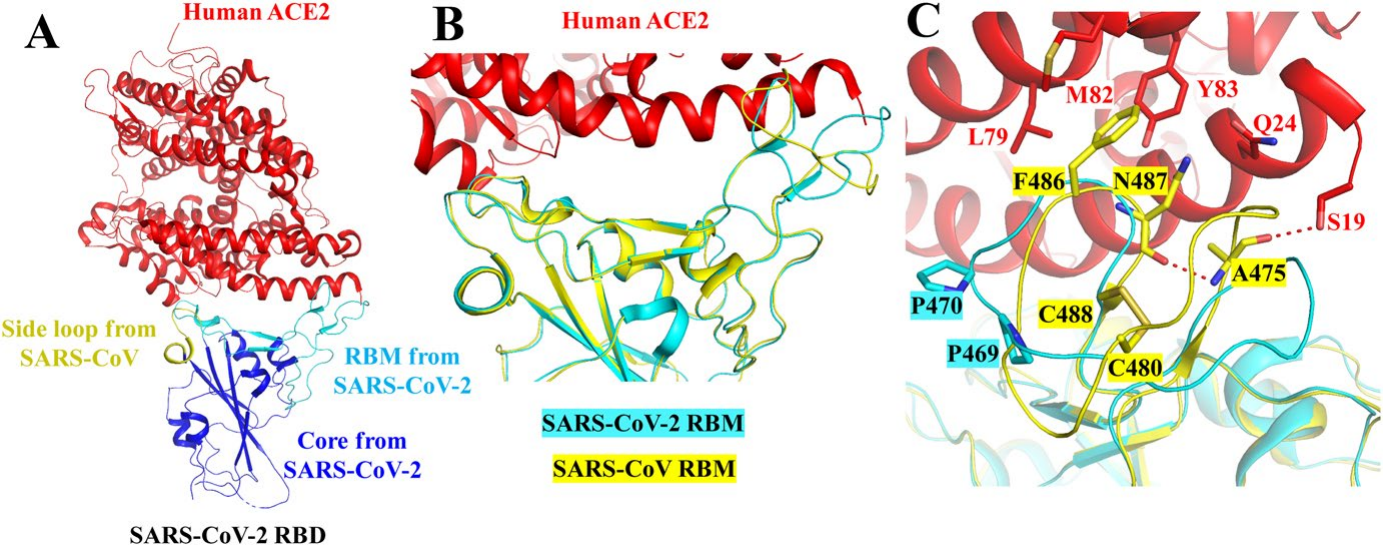
**a** The co-crystal structures of SARS-CoV-2 RBD in complex with ACE2. **b** Superposition of the ridge in SARS-CoV RBM (yellow) and SARS-CoV-2 RBM (cyan). **c** Superposition of the ridge from another visual angle (PDB ID 6VW1) [33]

Commonly expressed in lungs, intestine, kidneys and heart, ACE2 is a type I membrane protein [25–27]. Full-length *h*ACE2 consists of a carboxy terminal collectrin-like domain (CLD) and an amino terminal peptidase domain [26, 28]. However, the monoclonal antibodies that once were effective against SARS-CoV RBD do not neutralize SARS-CoV-2 and block virus infection because of some structural difference between two RBDs from SARS-CoV-2 and SARS-CoV [15, 29]. B^0^AT1 as the gene mutated in Hartnup disorder is a neutral amino acid (NAA) transporter that regulates the transport of NAAs into intestinal cells in a Na^+^-dependent manner. Reported evidence shows that B^0^AT1 employs ACE2 as a chaperone protein to achieve membrane trafficking [30]. However, it is unclear how B^0^AT1 interacts with ACE2. Recently, on the basis of the assumption that the structure of full-length ACE2 may be disclosed in the presence of B^0^AT1, Yan et al. reported the 3D structure of the *h*ACE2-B^0^AT1 complex and the RBD-ACE2-B^0^AT1 ternary complex that provides the structural basis for understanding SARS-CoV-2 recognition and infection by *h*ACE2 [31]. To reveal these differences and solve this problem, Wang, Li and co-workers determined the co-crystal structures of SARS-CoV-2 RBD in complex with ACE2 [32, 33]. As shown in Fig. 4a, b, the entire binding region of SARS-CoV-2 RBD with ACE2 is nearly congruent with that of SARS-CoV RBD and ACE2. The detailed structural analysis of SARS-CoV-2 RBD reveals that the majority of residues involved in binding either RBD are highly conserved, and the functionalities involved in side chain attachments in SARS-CoV-2 are nearly coincident with those in the SARS-CoV RBD [32]. Extensive studies show that SARS-CoV RBD contains a receptor-binding motif (RBM) and a core [13]. The contact interface between SARS-CoV-2 RBM and ACE2 is broader and tighter than that between SARS-CoV RBM and ACE2 [33].

Shang et al. confirmed that the significant structural change between RBMs from SARS-CoV-2 and SARS-CoV is the conformation of the loops in the binding model of RBM with ACE2 receptor (Fig. 4b, c) [33]. Specifically, SARS-CoV RBM contains a P-P-A-residue motif in this loop; SARS-CoV-2 RBM, by contrast, contains a G-V-E-G-residue motif; the two kinds of residue motifs allow the loop to adopt a distinct conformation (Fig. 4c). Additionally, there is a hydrogen bond (H bond) between Ala475 and Asn487 in the main chain of SARS-CoV-2 RBM, and hydrophobic interaction was formed by the Phe486 of SARS-CoV-2 RBM with residues Tyr83, Leu79 and Met82 of *h*ACE2. In summary, these structural traits of SARS-CoV-2 RBM are characterized by a higher binding affinity to ACE2 than seen for SARS-CoV.

The results of previous studies indicate that the activation of the envelope glycoproteins of various viruses, including Ebola virus, MERS-CoV, influenza virus and SARS-CoV, is mediated by the host cell protease [34, 35]. Hoffmann and colleagues demonstrated that the cleavage and priming of the S protein of SARS-CoV-2 that is employed for virus-host membrane fusion and cell entry is mediated by TMPRSS2 [18]. Cells, such as sustentacular cells of the olfactory epithelium, susceptible to SARS-CoV-2 virus infection have high ACE2 and TMPRSS2 expression [36]. Theoretically, the prevention of SARS-CoV-2 virus cell entry could be achieved by inhibiting TMPRSS2.

### Potential Drugs for Blocking the SARS-CoV-2 Cell Entry

ACE2 and TMPRSS2 receptors are recruited by the SARS-CoV-2 S protein to enable virus entry into cells. Inhibitors that target these key proteins as well as S protein sites and block SARS-CoV-2 cell entry need to be found and tested for their effectiveness as therapeutic agents [18].

**Fig. 5:**
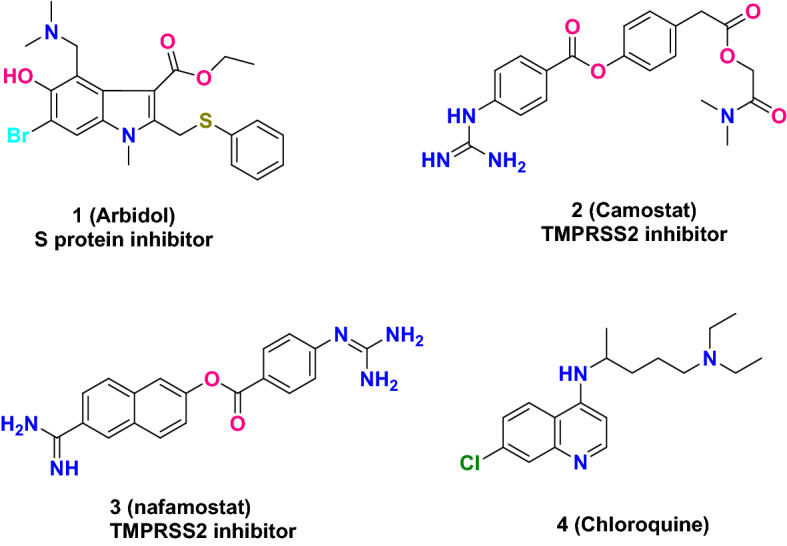
Reported compounds that block the SARS-CoV-2 cell entry

Arbidol (**1**, an indole-based small-molecule inhibitor), as shown in Fig. 5, is a broad-spectrum antiviral agent developed by the former Soviet Union Medical Chemistry Research Center. It inhibits cell entry of enveloped viruses by blocking virus-host cell membrane fusion [37–39]. Li's group reported that Arbidol inhibits SARS-CoV-2 cell entry in in vitro experiments. Molecular docking studies showed that it may do so by targeting S protein [16].

The serine protease inhibitor camostat (**2**, Fig. 5) is approved in Japan (1985) for treatment of chronic viral infections [34, 40], oral squamous cell carcinoma [41] and chronic pancreatitis [42]; it functions by targeting the serine protease TMPRSS2 that SARS-CoV-2 is reported to use for S protein priming [18]. It was found that camostat mesylate inhibits viral entry and reduces SARS-CoV-2 infection of Calu-3 lung cells [18]. Nafamostat (**3**) is another TMPRSS2 inhibitor approved in Japan for the treatment of acute pancreatitis, diffuse intravascular coagulation and anticoagulation in cardiopulmonary bypass [43, 44]. Nafamostat has been confirmed to block the membrane fusion of the virus with the lung Calu-3 host cell expressing TMPRSS2 by inhibiting the TMPRSS2 protease activity [45]. In view of the highly conserved amino acid sequence in S proteins of MERS-CoV and SARS-CoV-2, Xiao and co-workers [46] demonstrated that nafamostat exhibited good inhibitory activity against SARS-CoV-2 infection with EC_50_ of 22.50 μM, indicating that nafamostat as a TMPRSS2 inhibitor can prevent SARS-CoV-2 infection.

Chloroquine (**4**, Fig. 5), initially developed for the treatment of malaria and inflammatory (e.g., rheumatoid) arthritis [47], was reported to be highly effective in the management of SARS-CoV-2 infection in vitro by interfering with ACE2 [48]. The latest clinical trials carried out in China in COVID-19 patients show that chloroquine effectively shortens the time course of the disease and reduces deterioration of lungs due to pneumonia with acceptable safety constraints [49]. Although the in vitro inhibitory effect of chloroquine on several viruses including SARS-CoV-2 and its multiple mechanisms of action have been elucidated [50], its clinical pharmacology is still unclear [51]. The death of a man in Arizona reported by the New York Times [52] and the serious adverse reactions [53] induced a scientific debate engaged by several scientists on the effective use of chloroquine in the prevention and treatment of COVID-19. At present, there are no studies to evaluate the use of chloroquine for prophylaxis, and there is no convincing evidence to prove its significant clinical efficacy [51]. Accordingly, large, randomized, double-blind clinical trials with proper monitoring are required to determine whether chloroquine can be used as a powerful weapon to prevent and treat COVID-19 and has acceptable safety.

## RNA-Dependent RNA Polymerase (RdRp)

RNA-dependent RNA polymerase [RdRp, also referred to as nonstructural protein 12 (nsp12)] plays an important role in the synthesis of viral RNA from RNA templates, and especially in the replication and transcription of coronaviruses [54]. Therefore, RdRp has been the preferred target for the treatment of viral infection, such as SARS, MERS and chronic hepatitis viruses [55]. Recent sequence alignment studies have revealed that the SARS-CoV-2 RdRp shares highly homologous sequences with SARS-CoV RdRp [54]. Because blocking RdRp activity in SARS-CoV has been historically successful, it has also rapidly emerged as a promising therapeutic approach to the treatment of COVID-19.

### Structure of RdRp

**Fig. 6:**
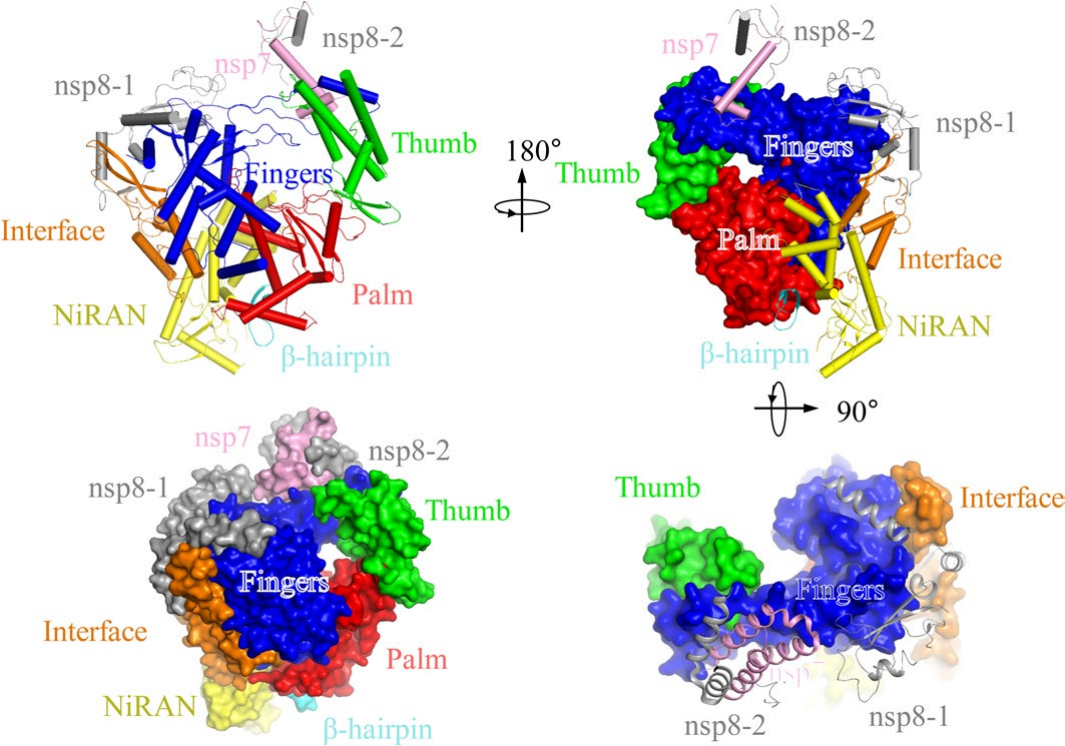
Cryo-EM structure of the nsp12-nsp7-nsp8 complex of SARS-CoV-2 (PDB ID 6M71) [56]

To facilitate the structure-based drug design, Rao et al. report the 3D structure of the SARS-CoV-2 full-length nsp12-nsp7-nsp8 complex at 2.9-Å resolution using cryo-EM (Fig. 6) [56]. This complex exhibited RNA polymerization activity in the presence of adenosine triphosphate (ATP) [57]. They also identified a newly discovered β hairpin domain at the amino termini of RdRp, in addition to the polymerase core that is conserved throughout the viral polymerase family. Overall, the architecture of the SARS-CoV-2 nsp12-nsp7-nsp8 complex is nearly identical with that of SARS-CoV [56]. The main difference between the RdRps from SARS-CoV-2 and SARS-CoV is the residue cluster Ala4-Arg118 and the residue cluster Asn215-Asp218, both of which are highly ordered in the SARS-CoV-2 nsp12 but less ordered in SARS-CoV nsp12 [56]. Qi Peng et al. reported the reduced polymerase activity and thermostability of the SARS-CoV-2 nsp12-nsp7-nsp8 core polymerase complex compared to that of SARS-CoV through 3D structure comparison, and this feature might contribute to the fitness of SARS-CoV-2 to human hosts [58]. Hillen et al. presented a 3D structure of the SARS-CoV-2 RdRp in an active form that mimics the replicating enzyme. The complex contains the viral proteins nsp12, nsp8 and nsp7, and more than two turns of RNA template–product duplex, while the two copies of nsp8 are mainly responsible for the binding with the opposite sides of the cleft and position of the second turn of RNA. The long helical extensions in nsp8 protrude along the exiting RNA, forming positively charged “sliding poles”, which could account for the known processivity of RdRp that is required for replicating the long genome of coronaviruses [59]. Liming Yan et al. determined the SARS-CoV-2 replication and transcription complex (RTC) which consisted of a template-primer RNA, nsp7 and nsp8, and two helicase molecules (nsp13-1 and nsp13-2) and revealed that the nsp13-1 motif is essential for the enhancement of the helicase activity of mini RTC [60].

**Fig. 7:**
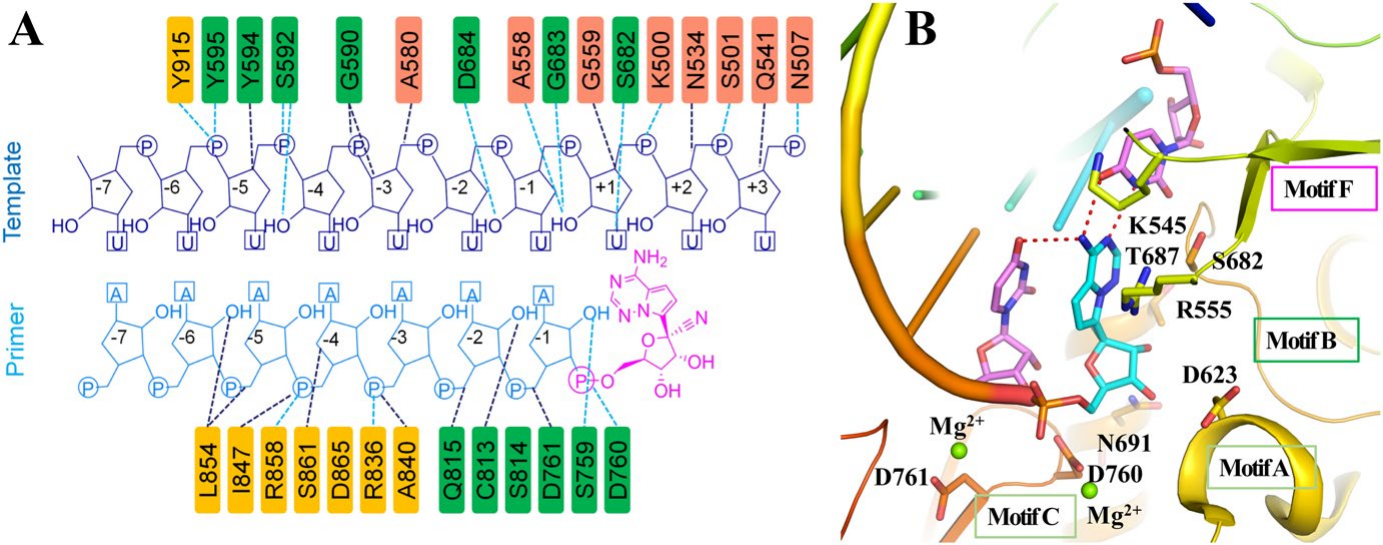
**a** Remdesivir monophosphate is covalently combined with the primer; **b** X-ray co-crystal structure of RNA bound RdRp in complex with remdesivir (PDB ID 7BV2) [57]

**Fig. 8:**
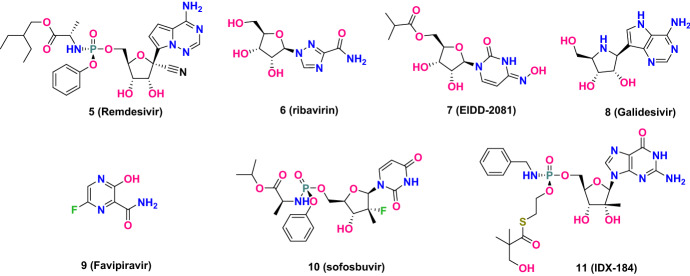
The structures of representative RdRp inhibitors

Structures of a template-primer RNA bound to SARS-CoV-2 RdRp and in complex with the antiviral drug remdesivir (**5**, GS-5734, Fig. 8) were determined using cryo-EM structural studies by Yin and co-workers [57]. Remdesivir is a prodrug that needs to be converted to the active triphosphate form [remdesivir triphosphate (RTP)] within the cells. It then covalently combines with the active center at the catalytic site of RdRp (the primer located at the +1 position in Fig. 7a) in the form of a nucleoside triphosphate active metabolite and indirectly inhibits viral RdRp transcription activity through strand termination at arbitrary positions of the template sequence. The backbones of RNA phosphate-ribose are involved in most protein-RNA interactions that directly involve 2′-OH groups (Fig. 7a). The RNA polymerization activity was completely inhibited by 1 mM RTP despite the exposure to physiological concentrations of ATP (10 mM) [57].

### Drug Repurposing for Treatment of COVID-19 by Inhibiting RdRp

The ongoing SARS-CoV-2 infection highlights the significance and urgency of antiviral agents [55]. Among several antiviral drugs, remdesivir, reported as an anti-Ebola virus inhibitor, is one of the most promising and hopeful antiviral agents used to control SARS-CoV-2 replication [61–63]. Remdesivir is a nucleoside analogue with broad-spectrum antiviral efficacy in HAE cells. The half-maximal effective concentration (EC_50_ value) for SARS-CoV and MERS-CoV are 69 nM and 74 nM, respectively [46, 63]. Unexpectedly, remdesivir less efficiently inhibits SARS-CoV-2 replication with an EC_50_ value of 0.77 μM. The co-crystal structure of RNA bound to RdRp in complex with remdesivir illustrates that the adenosine analogue of remdesivir monophosphate forms two H bonds with a uridine base from the template strand and additional base-stacking interactions with upstream adenosine bases from the primer strand (Fig. 7b) [57].

**Fig. 9:**
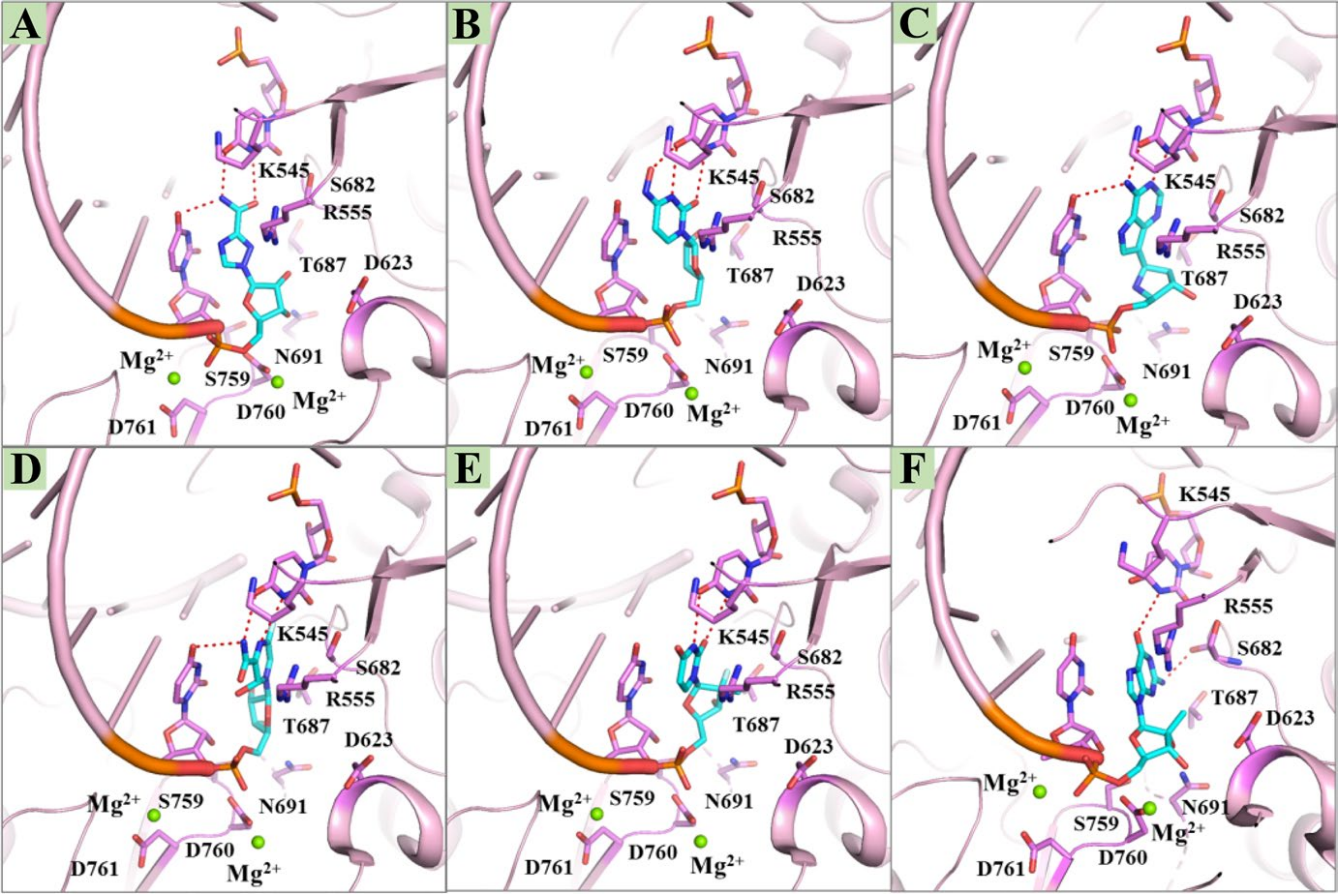
The proposed binding modes of inhibitors [ribavirin (**a**), EIDD-2081 (**b**), galidesivir (**c**), favipiravir (**d**), sofosbuvir (**e**) and IDX-184 (**f**)] with RdRp (PDB ID 7BV2)

As shown in Fig. 8, like remdesivir, other RdRp-targeted inhibitors, such as ribavirin, EIDD-2081, galidesivir, favipiravir, sofosbuvir and IDX-184, are all nucleotide analogues. They have been shown to be efficient in inhibiting viral RdRp in their triphosphate active forms [57, 64]. Figure 9 shows the molecular docking calculations performed by our group, which indicates that their binding configurations are nearly the same as remdesivir; i.e., they all covalently bind to RdRp in a ribosyl monophosphate form at the +1 position of the primer strand. Ribavirin is a broad-spectrum antiviral drug prescribed for the treatment of diseases caused by viral infections, such as hepatitis C and some viral hemorrhagic fevers. The antiviral activity against SARS-CoV was estimated to be at a concentration of 50 mg/ML in vitro [61, 65–68]. EIDD-2801 bearing good oral bioavailability is a prodrug of EIDD-1931 that has wide-spectrum antiviral activity against influenza virus and multiple coronaviruses, such as MERS-CoV, SARS-CoV and SARS-CoV-2. Specifically, the potency of EIDD-2801 in preventing SARS-CoV-2 replication is 3–10 times stronger than that of remdesivir [57, 69, 70]. This may be due to the two extra H bonds formed by the 4-hydroxyimino group of the cytidine ring with Lys545 and the cytidine base with the guanine base (Fig. 9b) [57]. Galidesivir (BCX4430) is an adenine analogue originally developed to treat hepatitis C virus. It is currently undergoing safety testing in early clinical studies and evaluation of its effectiveness in treating yellow fever. In preclinical studies, it has shown activity against a variety of RNA viruses, including SARS and MERS [71].

Of the pyrimidine analogues, favipiravir (T-705) is also a novel RdRp-targeted inhibitor that is initially phosphoribosylated by enzymes and then converted to favipiravir-ribofuranosyl-5′-triphosphate (an active conformation) that exhibits influenza RdRp inhibitory activity with a half-maximal inhibitory concentration (IC_50_) of 341 nM. Recently, preliminary clinical trials have shown favipiravir has encouraging anti-polymerase activity for use in treatment of patients infected by SARS-CoV-2 [57, 71–73].

Sofosbuvir (PSI-7977) and IDX-184 are HCV RNA replication inhibitors. Using docking data, Elfiky et al. showed that IDX-184 and sofosbuvir tightly bind to the SARS-CoV-2 RdRp. They suggested that further optimization of these two compounds could result in a more potent compound that may be effective against SARS-CoV-2 [74–76].

## The Main Protease (M^pro^, Also Named a Chymotrypsin-Like Cysteine Protease, 3CL^pro^)

According to recent research [77, 78], two polyproteins (pp1a and pp1ab) in SARS-CoV-2 are coded in the two open reading frames, ORF1a and ORF1b, that consist of approximately two-thirds of the entire genome length. Subsequently, the two polyproteins are processed into various nonstructural proteins by employing the two viral-borne proteases, M^pro^ and PL^pro^ (papain-like protease). That the role of M^pro^ is vital in adjusting coronavirus replication and transcription of the viral life cycle has been demonstrated in two major studies [79, 80]. The homologous protein of M^pro^ does not exist in humans. Therefore, the possibility of targeting M^pro^ by various therapeutic reagents has been viewed as a promising strategy for the treatment of coronavirus diseases. Efforts to target M^pro^ have been given considerable attention from numerous researchers [81, 82].

At present, the detailed catalytic mechanism of SARS-CoV-2 M^pro^ action is still unknown. Because it belongs to the β-coronavirus family, SARS-CoV-2 M^pro^ may share similar catalytic mechanisms with SARS-CoV M^pro^ and MERS-CoV M^pro^. As previously reported, SARS-CoV M^pro^ and MERS-CoV M^pro^ cleave the polyproteins that generally favor Leu-Gln↓(Ser, Ala, Gly) sequence sites in the manner of a nucleophile [83–87]. Recently, Shang et al. [88] showed that MERS-CoV M^pro^ and SARS-CoV M^pro^ exploit the existence of a partial negative charge cluster (Arg-Tyr-Asp) and a conserved water molecule to accelerate catalysis. They inferred the existence of a partial negative charge cluster (PNCC) at the active interface and suggested it become a potential target for interposing a blocking agent that cancels the dipole moment of PNCC in the prospect that it inhibits proteolytic activity. Following the widening COVID-19 pandemic, an increasing number of groups from all over the globe are testing drug candidates that target SARS-CoV-2 M^pro^.

### The Structure of SARS-CoV-2 M^pro^

**Fig. 10:**
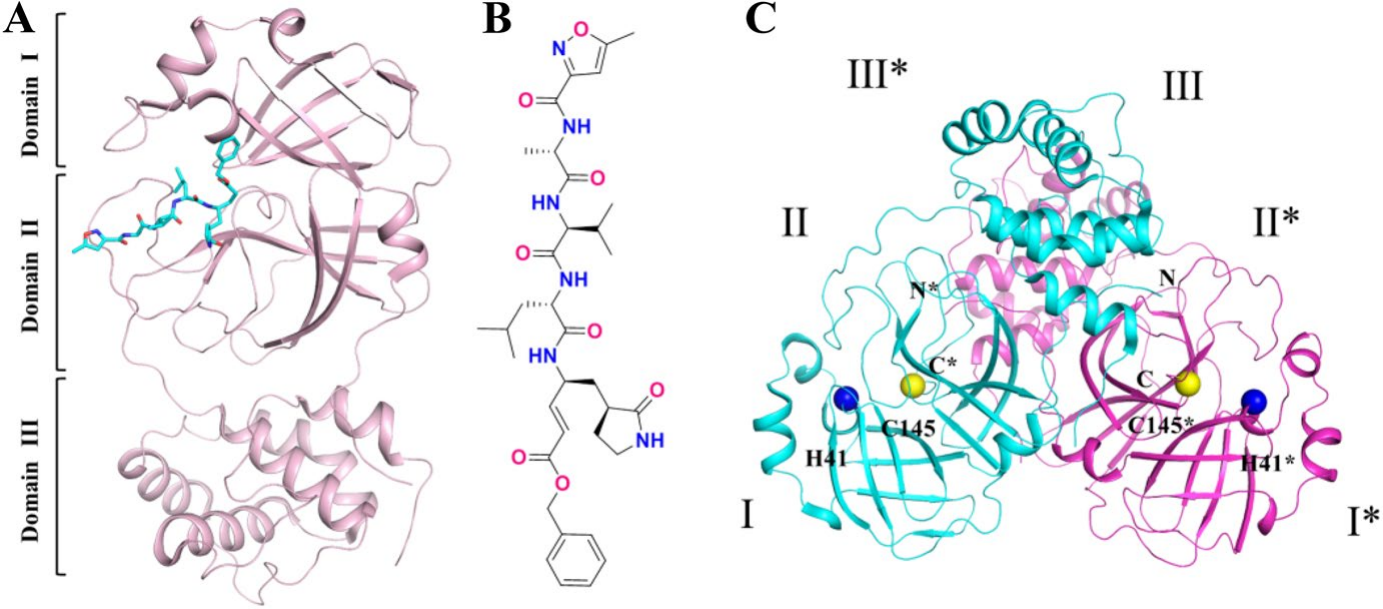
**a** The structure of one monomer of the dimeric SARS-CoV-2 M^pro^ with inhibitor N3 (PDB ID 6LU7) [82]; **b** the chemical structure of **N3** inhibitor; **c** the inhibitor-free crystal structure of the dimeric SARS-CoV-2 M^pro^ (PDB ID 6Y2E) [89]

The first crystal structure of SARS-CoV-2 M^pro^ was examined at a 2.1-Å resolution after co-crystallization with the inhibitor **N3** (Fig. 10a, b) [82]. Subsequently, structures of its inhibitor-free form, as well as an α-ketoamide inhibitor-co-crystalized form, were also made available (Fig. 10c) [89]. The M^pro^ of SARS-CoV-2 exists as a homodimer, which is consistent with the composition of most known coronaviruses [82]. As shown in Fig. 10a, each monomer is composed of three domains (domains I–III). Domains I and II involve residues 8–101 and residues 102–184, respectively. They comprise six-stranded antiparallel β-barrels. Domain III is linked to domain II via a long loop region (residues 185–200) and consists of a globular cluster of five α-helices that are closely related to the dimerization of the M^pro^. The protease catalytic cavity occupies the cleft between domains I and II. There are two deep-buried subsites (S1 and S1′) and three shallow subsites (S2-S4) in the pocket [90], and their roles in binding mode will be described in detail in the following. It is worth noting that an active dyad Cys145-His41 (His41 serves as a proton acceptor, and Cys145 serves as the nucleophile) located in the cavity is highly conserved in the M^pro^ from other coronaviruses (Fig. 10c) [91–94].

Protein sequence alignment of M^pro^ in SARS-CoV [95] and SARS-CoV-2 [89] indicates up to 96% amino acid sequence homology with only 12 different residues between the two proteases. The residue Cys145 that forms a C–S covalent bond with inhibitors is a key site in the substrate-binding pocket of M^pro^ [96]. The site is absolutely conserved in both proteases. In view of the above, repurposing of protease inhibitors that bind to Cys145 of SARS-CoV M^pro^ may be a promising approach for treatment of COVID-19 to meet the great challenge to discover effective and specific antiviral drugs. It is worth mentioning that the Ser46 residue at the active site of SARS-CoV-2 M^pro^ may influence the behavior of the substrate-binding pocket and enable novel blocking interactions with protease inhibitors.

By superposition of their crystal structures, Jin et al. demonstrated that the substrate-binding pockets for the M^pro^ proteases for the 12 reported coronaviruses are highly conserved. This suggests that the previously tested drugs that target this pocket may be useful as broad-spectrum antiviral inhibitors [82, 97]. Thus, the repurposing of already-approved antiviral agents or antiviral drug candidates in various stages of clinical trials may become a feasible approach to speed discovery of effective drugs to control the spread of COVID-19. Previous investments in time and money may help to compensate for the time-consuming and high costs of conventional drug development.

### Inhibitors Targeting SARS-CoV-2 M^pro^ and the Binding Modes Between Them

**Fig. 11:**
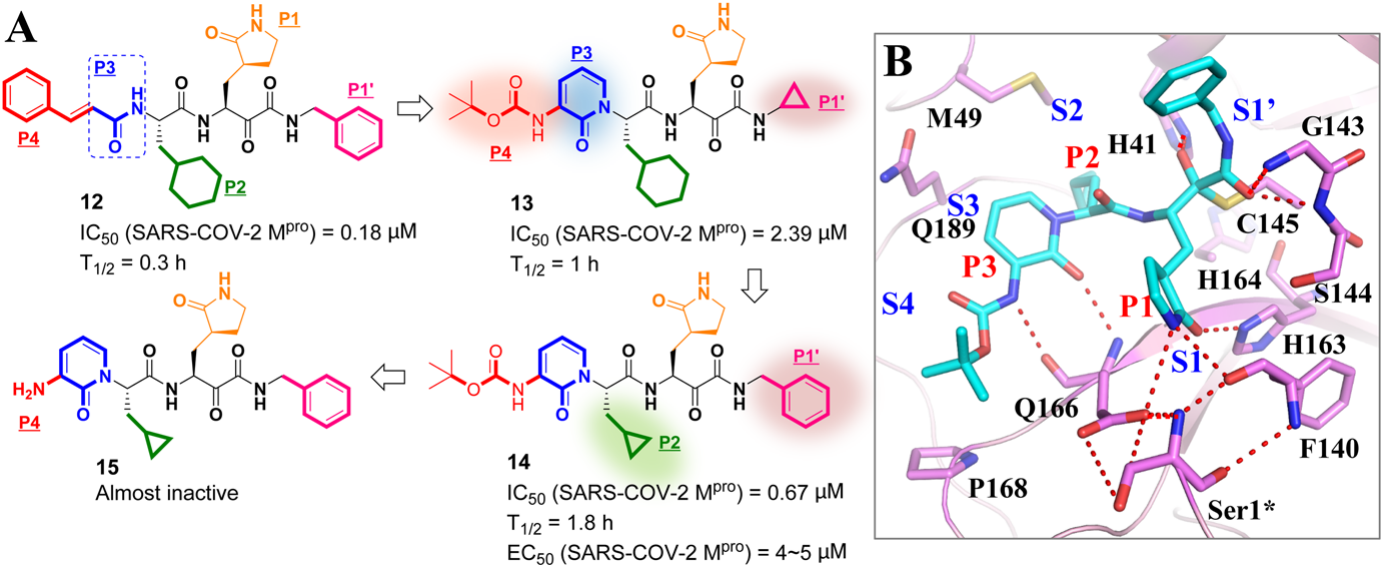
**a** Structures of α-ketoamide inhibitors **12**, **13**, **14** and **15** (the modification process is highlighted with background color); **b** co-crystal structure of inhibitor **14** in the active site of SARS-CoV-2 M^pro^ (PDB: 6Y2F) [89]

Although there is a dearth of specific antiviral drugs to mitigate the COVID-19 epidemic, several drug candidates having encouraging clinical potential and have been described by numerous investigators. Zhang and co-workers discovered a peptidomimetic α-ketoamide analogue **12** (see Fig. 11a) with picomolar level inhibitory activity (EC_50_ of 400 pM) against MERS-CoV and low-micromolar inhibition (EC_50_ value in μM) against SARS-CoV in cell-based assays [98]. Given the highly conserved substrate-recognition pocket in two M^pro^s from SARS-CoV-2 and SARS-CoV, the same authors treat **12** as a reasonable starting point for exploration of novel SARS-CoV-2 M^pro^ inhibitors. Impeded by the poor metabolic stability (T_1/2_ = 0.3 h) of **12**, they initially developed structural modifications on analogue **12** to improve its half-life in plasma [89]. Replacing the styryl group in the P4 moiety with a *tert*-butyloxycarbonyl protecting group (Boc-NH) that protects the amide bond in the P3 moiety with a pyridone, and converting the benzyl group in the P1′ moiety to a cyclopropyl group, these changes generated significant improvement (~ three fold) in the plasma half-life in mice of compound **13** (T_1/2_ = 1 h), relative to compound **12**. However, they also resulted in approximately 13.3-fold loss of inhibitory activity (IC_50_ value reduced from 0.18 to 2.39 μM). To restore the antiviral activity of **13**, a smaller cyclopropyl group was introduced in the P2 moiety to replace the cyclohexyl functionality that appears extensively in a number of broad-spectrum inhibitors of M^pro^. In addition, the newly substituted cyclopropyl group in the P1′ moiety was returned to a benzyl group. The resulting compound **14** was able to inhibit SARS-CoV-2 M^pro^ at sub-micromolar concentrations (IC_50_ = 0.67 μM). In addition, it possessed longer half-life when administrated systemically in mice (T_1/2_ = 1.8 h). Importantly, compound **14** showed favorable antiviral efficacy in SARS-CoV-2-infected Calu3 cells in an in vitro model with an EC_50_ value of 4–5 μM. However, upon deprotecting the amine function in moiety P4, compound **15** was formed and was almost completely inactive. In addition, compound **15** showed favorable absorption, distribution, metabolism and excretion (ADME) properties and lung tropism, stimulating the development of the pyridone-containing inhibitors for anti-COVID-19 drugs.

As shown in Fig. 11b, compound **14** locates in the substrate-recognition pocket of SARS-CoV-2 M^pro^ in an extended conformation. The S1 subsite, composed of His163, Phe140, His172, Cys145 and Glu166 residues, securely anchors the P1 γ-lactam moiety via three H bonds linking between the lactam and Phe140, Glu166 and His163. In addition, the α-ketoamide moiety in **14** was expected to target residue Cys145 to form a stable C–S covalent bond. The benzyl group in the P1′ moiety extends into the bulky S1′ subsite that consists of Cys145, Gly143, His41 and Thr25 residues to interact with Gly143, His41 and Thr25 by van der Waals interaction. The hydrophobic S2 subsite, bearing a stereoscopic volume of 28 Å^3^ occupied by Met165, Met49 and His41 residues, suitably accommodates the cyclopropylmethyl group in P2. It is worth mentioning that the S2 pocket (with a stereoscopic volume of 252 Å^3^) in SARS-CoV M^pro^ is larger than the corresponding S2 pocket of SARS-CoV-2 M^pro^, enabling it to adapt to the larger bulky cyclohexylmethyl group in P2 of compound **12** [98]. The pyridone ring in P3 of **14** occupies the S3 subsite formed by the side chains of Glu166 and Gln189 that is stabilized with a H bond between the carbonyl oxygen of pyridine and the main-chain amide of the Glu166 residue. The protecting Boc-NH group in P4 faces towards Pro168, but does not occupy the authorized S4 subsite of the protease.

**Fig. 12:**
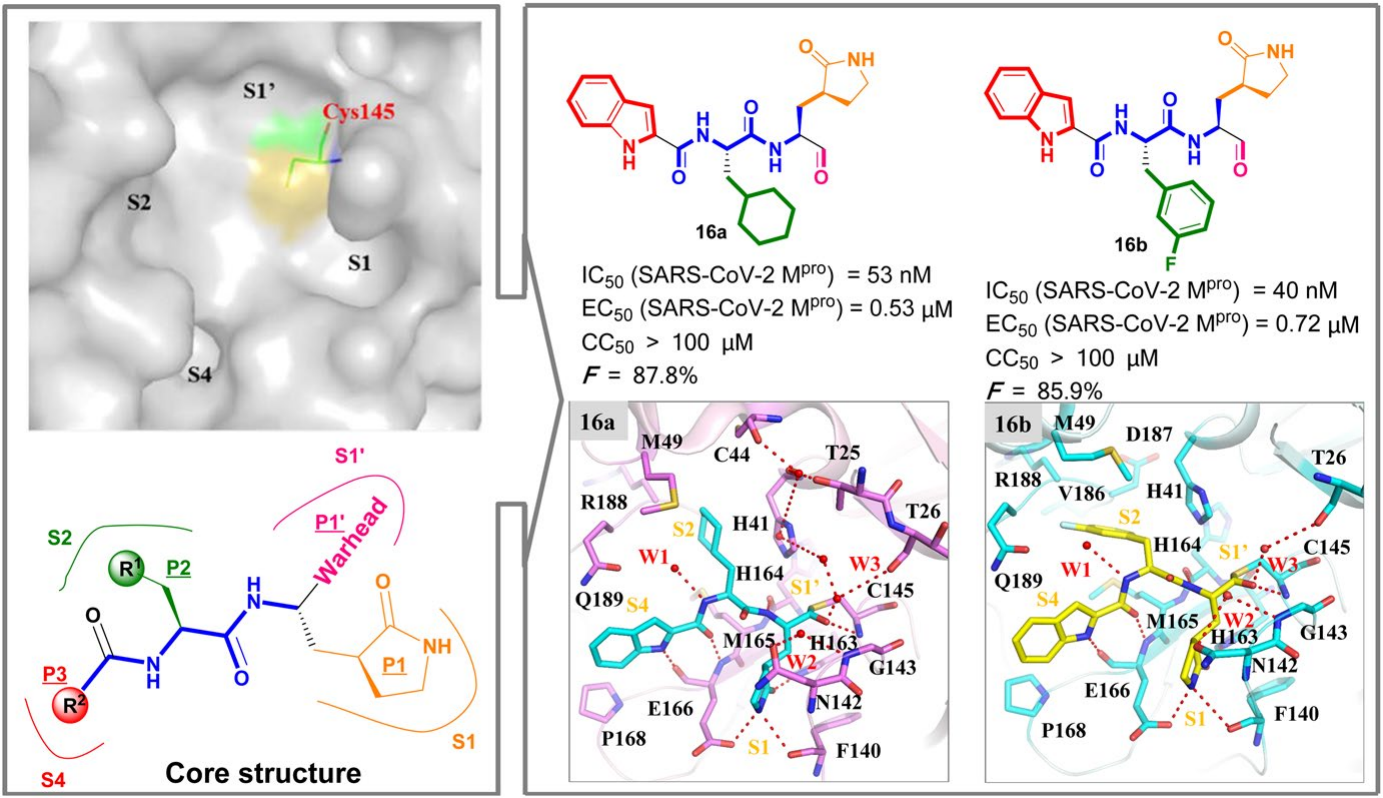
Medicinal chemistry information of novel peptidomimetic-based inhibitors **16a–b** against SARS-CoV-2 M^pro^ from design to candidates

Based on the substrate-recognition pocket of homologous virus SARS-CoV M^pro^ (Fig. 12), Dai et al. recently reported two novel peptide-mimetic-based lead compounds (**15a** and **15b**) for SARS-CoV-2 M^pro^ [99]. On the basis of the core structure of the peptidomimetic covalent inhibitor, a number of privileged groups have been inserted into reported M^pro^ inhibitors, including:

1. An aldehyde group expected as a warhead to form a C–S bond with the Cys145 residue;
2. A cyclohexyl or 3-fluorophenyl group that is expected to insert deeply into the S2 subsite; and
3. An indole group expected to form new H bonds with S4 subsite.

These were introduced into P1′ (pink mark), P2 (green mark) and P3 (red mark) moieties, respectively, when the fixed (*S*)-γ-lactam ring was substituted as the P1 moiety. Subsequently, extensive in vitro and in vivo studies led to the identification of the antiviral drug candidates **16a** and **16b** (in Fig. 12), which demonstrated excellent in vitro potency (IC_50, SARS-CoV-2 Mpro_ of 53 nM and 40 nM, respectively) and good bioavailability (fraction of dose *F* achieving systemic circulation of 87.8% and 85.9%, respectively). Compounds **16a** and **16b** displayed excellent cell-based antiviral efficacy (EC_50_ = 0.53 μM, and 0.72 μM, respectively) with acceptable cytotoxicity (CC_50_ > 100 μM). In co-crystalized structures of **16a** (PDB ID 6LZE) and **16b** (PDB ID 6M0K) with SARS-CoV-2 M^pro^, both inhibitors were securely anchored in the substrate-binding pocket through the C–S covalent bond with the Cys145 residue. They were further stabilized by an H bond between aldehyde groups and the Cys145 residue. In addition, the cyclohexyl or 3-fluorophenyl group is inserted deeply into the S2 subsite and held by *π*–*π* stacking and hydrophobic interactions. The indole group occupies the S4 subsite as expected.

**Fig. 13:**
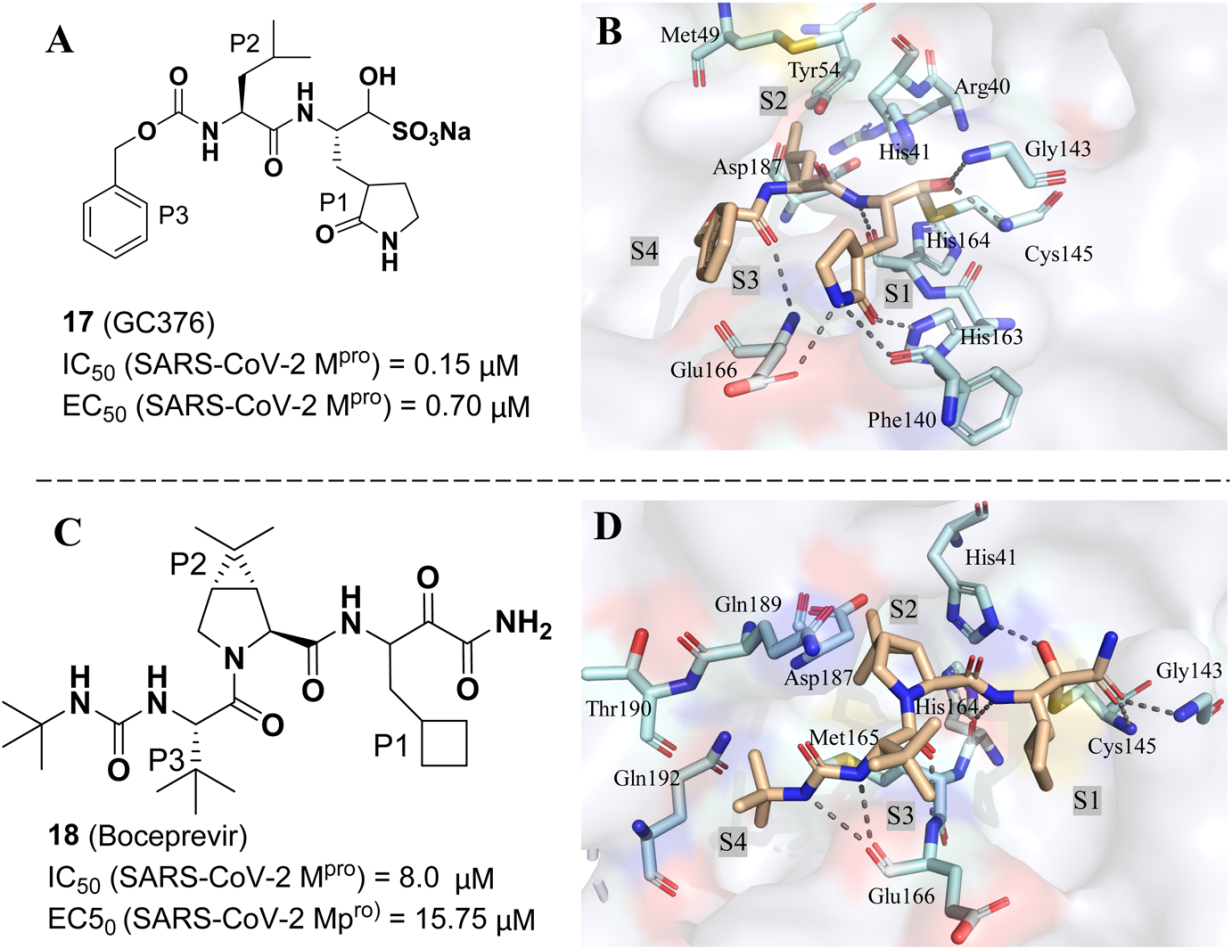
The structures of GC376 **a** and boceprevir **c**; Co-crystal structures of GC376 **b** (PDB ID: 7C6S) and boceprevir **d** (PDB ID: 7C6U) in the active site of SARS-CoV-2 M^pro^

In order to address unmet clinical need in COVID-19 therapy, Gao et al. recently described the biological activities of GC376 (**17**) and boceprevir (**18**) against M^pro^ and SARS-CoV-2 according to drug repurposing strategy (Fig. 13) [100]. As shown in Fig. 13, GC376, a broad-spectrum antiviral drug targeting cysteine protease, and boceprevir, an FDA-approved antiviral medication for the treatment of chronic hepatitis C, both can inhibit the SARS-CoV-2 M^pro^ activity well with IC_50_ values of 0.15 μM and 8.0 μM, respectively. Furthermore, GC376 and boceprevir displayed excellent anti-SARS-CoV-2 replication efficacy (EC_50_ = 0.70 μM, and 15.75 μM, respectively) with excellent selectivity over the bovine chymotrypsin and acceptable cytotoxicity. To interpret mechanisms of these two compounds against SARS-CoV-2 M^pro^, they determined the co-crystal structures of GC376 (Fig. 13b) and boceprevir (Fig. 13d) [100]. Both of them were tightly anchored in the substrate-binding pocket through the C–S covalent bond with the Cys145 residue and further stabilized by several H bonds and hydrophobic interactions between ligands and residues.

**Fig. 14:**
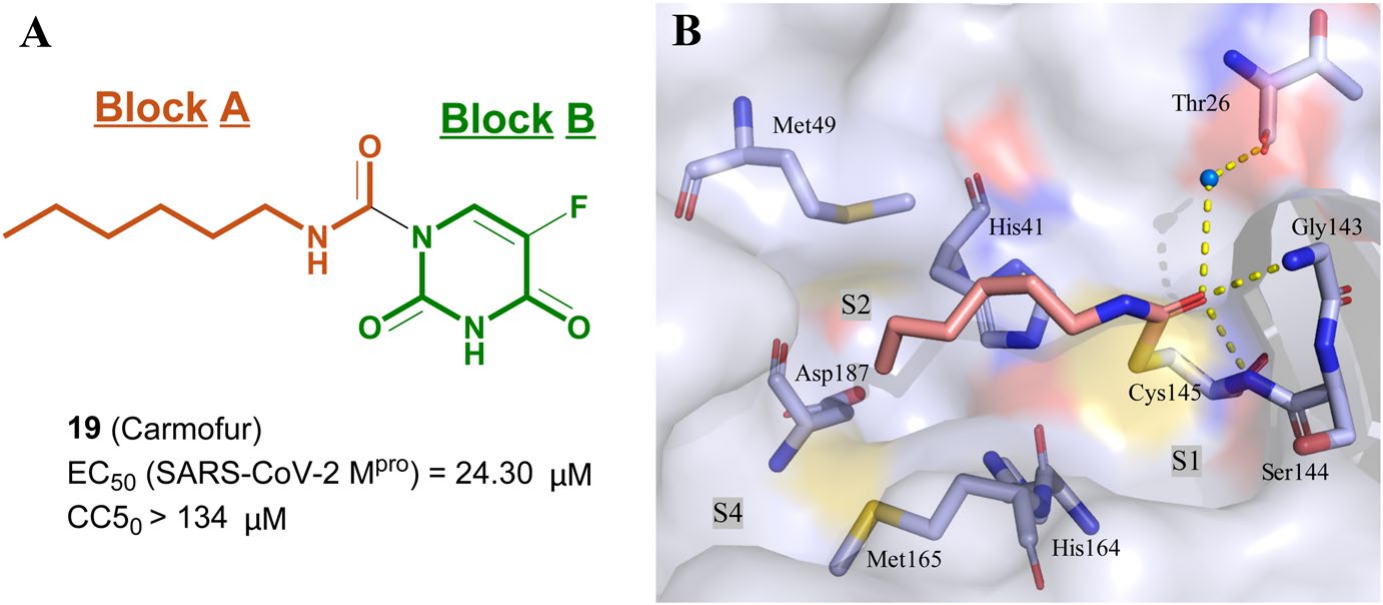
**a** The chemical structure of carmofur; **b** co-crystal structures of carmofur in the active site of SARS-CoV-2 M^pro^ (PDB ID: 7BUY)

Rao and colleagues identified that carmofur can be used as a promising lead compound to develop new anti-SARS-COV-2 drugs for treatment of COVID-19 (Fig. 14) [101]. Carmofur effectively inhibits SARS-CoV-2 replication in Vero cells (EC_50_ = 24.30 μM) with acceptable cytotoxicity (CC_50_ > 134 μM). The X-ray crystal structure of the carmofur/M^pro^ complex disclosed the elimination of pyrimidinedione (block B) and covalent modification of Cys145, which is caused by the sulfhydryl group of Cys145 attacking the carbonyl group in block A of carmofur. In addition, they observed that the residues Ser144 and Gly143 formed two hydrogen-bonding interactions with a carbonyl group in block A and a water bridge between oxygen atom of carbonyl in block A and Thr26. the fatty carbon chain tail is inserted deeply into the S2 subsite and held by hydrophobic interactions.

### Inhibitors Targeting SARS-CoV M^pro^

The development of homologous virus SARS-CoV M^pro^ inhibitors acts an important role in driving rapid discovery of SARS-CoV-2 M^pro^ inhibitors, given the great many highly conserved active sites of M^pro^ in all known coronaviruses, especially in SARS-CoV and SARS-CoV-2. Currently, the reported peptide-mimetic-based covalent inhibitors targeting SARS-CoV M^pro^ are typically designed with a chemical “warhead” [81, 102]. The current research status involving peptidomimetic-based SARS-CoV M^pro^ inhibitors can be summarized based on specifying different types of warheads. They include Michael acceptors, aldehydes, substituted ketones, keto-glutamines, epoxides and boronic acid.

**Fig. 15:**
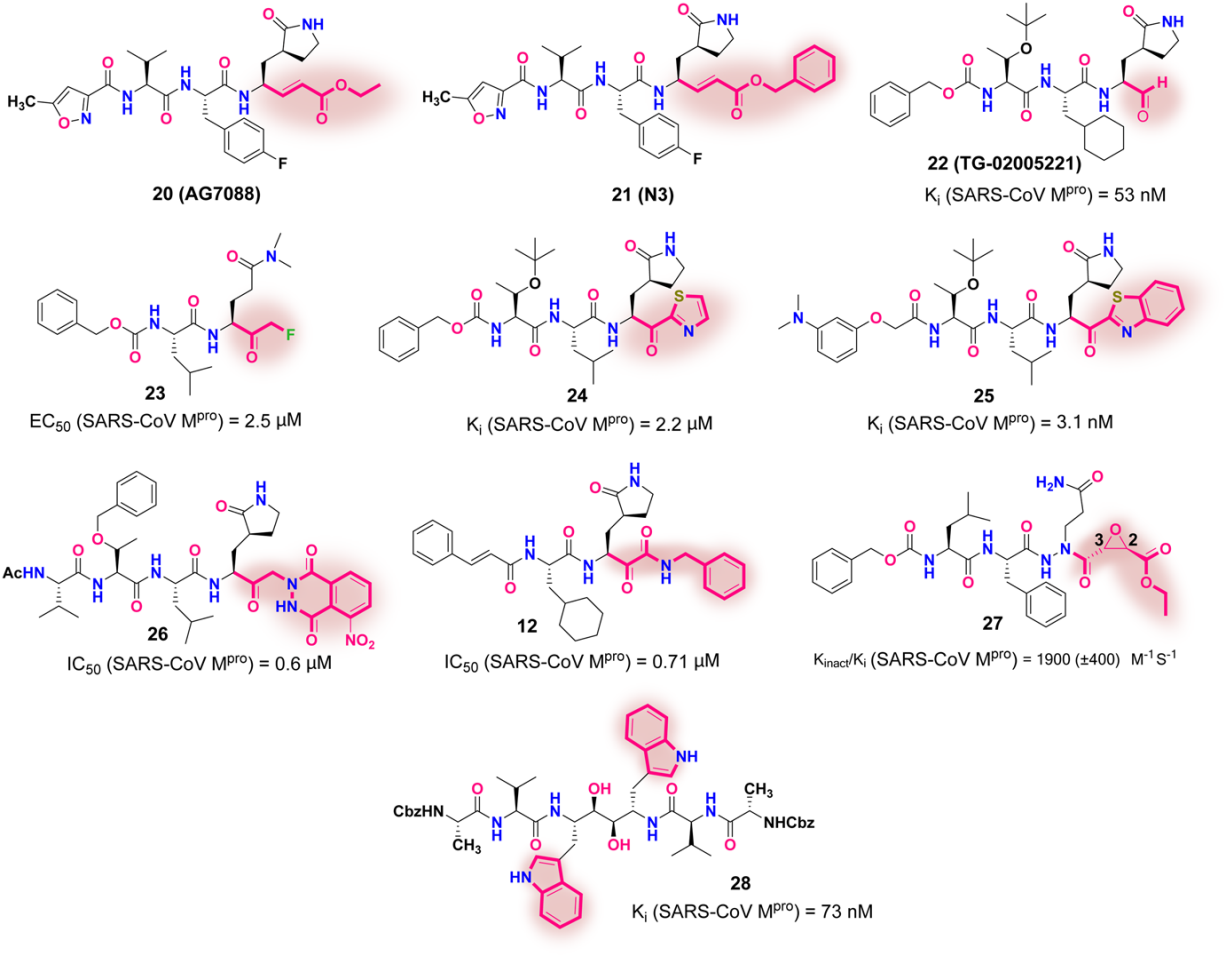
The structures of representative peptidomimetic inhibitors **20–28** incorporating various warheads (identified in pink clouds)

As shown in Fig. 15, AG7088 (**20**, rupintrivir), a potent ethoxy-containing Michael acceptor-based peptidomimetic inhibitor, has already been clinically studied for use against the common cold rhinovirus 3C protease (HRV2 3C^pr^°) [103]. AG7088 has been proposed as a starting point in the drug design of new SARS-CoV M^pro^ inhibitors [79, 104–106]. In 2005, Yang and co-workers identified a benzyloxy-containing compound **21** (N3, an analogue of AG7088, Fig. 15) with a Michael acceptor as a broad-spectrum anti-coronavirus inhibitor (e.g., SARS-CoV M^pro^ and MERS-CoV M^pro^) [82, 97].

Yang and colleagues disclosed a novel, potent aldehyde-based SARS-CoV M^pro^ inhibitor **22** (TG-02005221, Fig. 15) [107]. Compound **22** displayed robust antiviral activity with a K_i_ value of 53 nM as well as favorable pharmacokinetic profiles in rodents. The X-ray crystal structure of SARS-CoV M^pro^ in complex with **22** showed that compound **22** was tightly anchored on the active site of M^pro^ via a covalent C–S bond, together with 10 H bonds and an extensive assemblage of hydrophobic interactions (PDB ID 2GX4).

Cai and co-workers reported a series of new SARS-CoV M^pro^ inhibitors that employed a fluoromethyl ketone group as a new warhead, and also replaced the widely studied (*S*)-γ-lactam ring in the P1 moiety with a dimethylamino oxoethyl group (corresponding to the ring opening of the lactam) [108]. The most potent compound **23** (Fig. 15) showed good cell-based antiviral efficacy (EC_50_ = 2.5 μM) with low toxicity in mice.

Inspired by the electron-withdrawing aspects of the fluoromethyl ketone group in **23**, the effects of other types of electron-withdrawing groups (thiazolyl and benzothiazolyl) on anti-SARS-CoV M^pro^ replication activity were investigated by Hayashi and co-workers. They identified two lead compounds, **24** and **25** (Fig. 15), with potent antiviral efficacy [109–112]. Compound **24** brandishes a thiazolyl ketone warhead and (*S*)-γ-lactam at P1, showing favorable inhibitory activity against SARS-CoV M^pro^ (K_i_ = 2.2 μM, IC_50_ = 9.5 μM) [109]. Compound **25,** bearing a benzothiazolyl ketone warhead, exhibited excellent inhibitory activity with a K_i_ value of 3.1 nM [110]. The co-crystal structure of SARS-CoV M^pro^ in complex with **24** reveals that the ketone group of **24** is converted to an oxyanion after attack by the thiol of the Cys145 residue. As a result, the arch-reason for ketone as an acceptable warhead is the formation of multiple H bonds between the generated oxyanion and three M^pro^ residues: Cys145, Gly143 and Ser144. In addition, the nitrogen in the thiazole plays an important role in stabilizing the binding model of **24** with SARS-CoV M^pro^ by forming an H bond with the His41 residue.

In 2004, as the promising effects of glutamine analogues against hepatitis A virus (HAV) became evident [113, 114], Vederas and co-workers reported a series of phthalhydrazide-based keto-glutamine analogues as novel SARS-CoV M^pro^ inhibitors [115]. Compound **26** (Fig. 15) was identified as the most potent anti-SARS-CoV M^pro^ inhibitor with an IC_50_ of 0.6 μM [115]. Molecular modeling studies of **26** indicated that the bulky phthalhydrazide group was accommodated at the S1′ subsite of the enzyme and formed a key H bond between Asn142 and the nitro group attached to the phthalhydrazide. In light of the novel phthalhydrazide-containing ketone-based warhead of **26**, Zhang and co-workers proposed the introduction of an α-ketoamide warhead into the P1′ moiety based on de-cyclization of the phthalhydrazide group in **26** [98]. Extensive biological evaluation led to the broad-spectrum inhibitor **12** (mentioned in Fig. 11 above) that demonstrated inhibition of the activity of SARS-CoV M^pro^ with a low-micromolar IC_50_ value (0.71 μM). Moreover, further crystallographic study of **12** (PDB ID: 2BX4) demonstrated that the α-keto-carbon formed a hemithioacetal by a condensation reaction with the mercapto group of Cys145.

James et al. reported that a novel substrate-like aza-peptide inhibitor in compound **27** (Fig. 15) contained an epoxy structure with prominent activity [K_inact_/K_i_ (SARS-CoV M^pro^) = 1900 ± 400 M^−1^S^−1^] [116, 117]. The co-crystal structures of SARS-CoV M^pro^ in complex with **27** demonstrated the formation of a C–S covalent bond between the epoxide C-3 and sulfur atom in Cys145 (PDB ID 2A5K) [117]. Interestingly, a symmetric peptide-like inhibitor **28** (Fig. 15) [118] with a K_i_ value of 73 nM for SARS-CoV M^pro^ was disclosed based on in silico optimization of a noncovalent HIV protease inhibitor by Wong and co-workers [119].

**Fig. 16:**
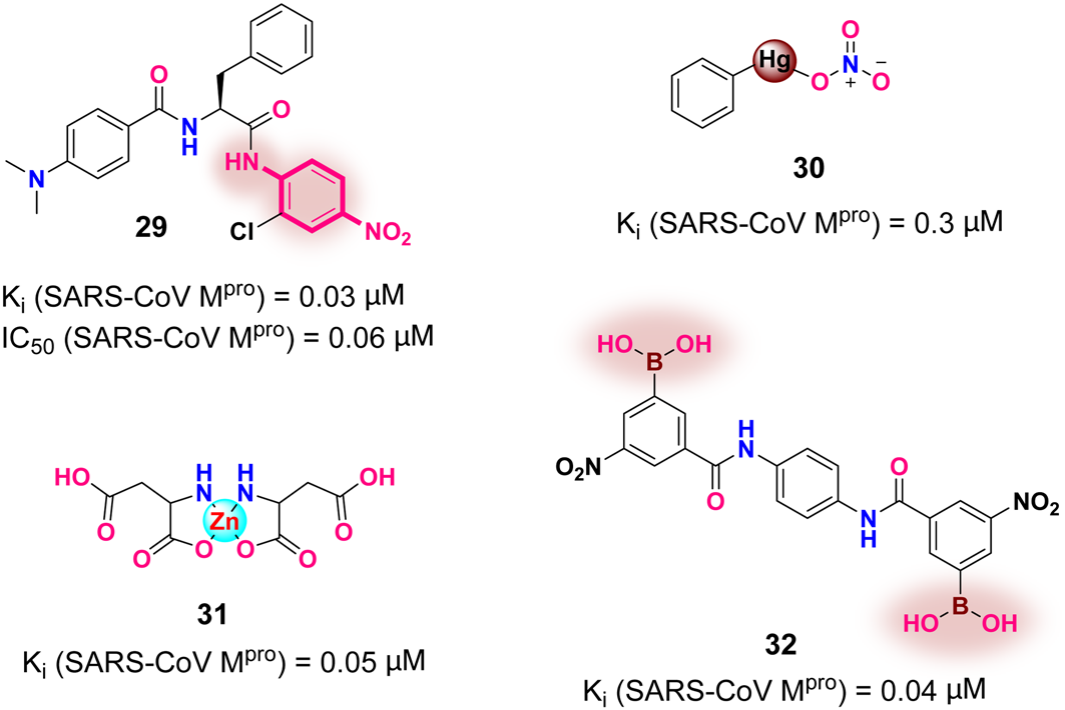
The structures of small-molecule inhibitors **29–32** against SARS-CoV M^pro^ (warheads are identified in pink clouds)

Besides the peptidomimetic inhibitors mentioned above, small-molecule inhibitors targeting SARS-CoV M^pro^ have also been extensively explored. Wong and co-workers produced a series of nitroaniline derivatives, among which compound **29** (Fig. 16) was discovered and functions as a competitive, noncovalent inhibitor targeting SARS-CoV M^pro^; it displays potent inhibitory efficacy (K_i_ and IC_50_ values of 0.03 and 0.06 μM, respectively) [120]. Currently, the increasing exploitation of metal-conjugated compounds as broad-spectrum antiviral agents has been widely investigated [121–124]. In one example, Hsu and co-workers discovered a mercury-conjugated inhibitor **30** (Fig. 16) that effectively inhibits the activity of SARS-CoV M^pro^ (K_i_ = 0.3 μM) by utilizing the affinity of the thiol group in Cys145 to Hg^2+^ ions [125]. Another example is the zinc-conjugated inhibitor **31** (Fig. 16) reported by Lee and co-workers [126]. Compound **31** displayed good antiviral activity with a K_i_ value of 0.05 μM by benefiting from the binding of Zn^2+^ ions to the His41-Cys145 catalytic dyad.

Bacha and co-workers [127] discovered an attractive subsite at the cluster of residues Ser147-Ser139-Ser144 that are close by the catalytic residues in SARS-CoV M^pro^. As the cluster of Ser residues in all reported coronavirus M^pro^ is highly conserved, this cluster may provide a common target site of broad-spectrum anti-coronavirus inhibitors [128]. Since the efficacy of aryl boronic acid derivatives against serine protease was demonstrated in the 1970s, a great number of boronic acid compounds have been investigated for use as protease inhibitors that feature both site selectivity and host safety [129, 130]. The recognized mechanism of inhibition is the production of a boronate-based tetra-coordinate complex by a nucleophilic coordination process between the –OH group in serine and boronic acid derivatives [128]. In view of the above recognized mechanism, a series of bifunctional boronic acid-conjugated SARS-CoV M^pro^ inhibitors were evaluated by Bacha et al., ultimately leading to the potent antiviral inhibitor **32** (Fig. 16) with a *K*_*i*_ value of 0.04 μM [127].

**Fig. 17:**
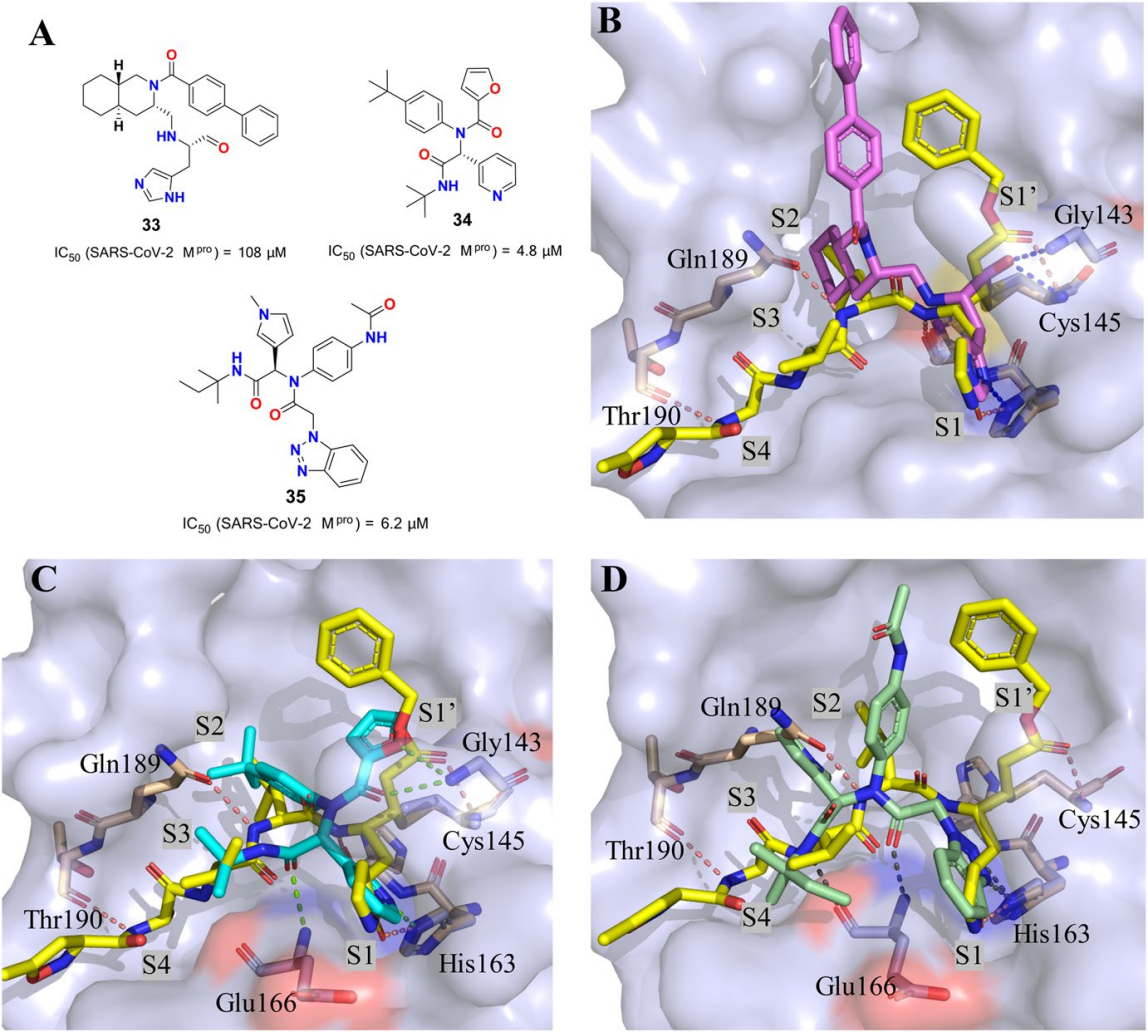
**a** The chemical structures of representative non-peptide SARS-CoV M^pro^ inhibitors; **b** superposition of co-crystal structures of **N3** (yellow, PDB ID: 2HOB) and compound **33** (violet, PDB ID: 4TWY) with SARS-CoV M^pro^; **c** superposition of co-crystal structures of **N3** (yellow) and compound **34** (cyan, PDB ID: 3V3M) with SARS-CoV M^pro^; **d** superposition of co-crystal structures of **N3** (yellow) and compound **35** (pale green, PDB ID: 4MDS) with SARS-CoV M^pro^. The H bonds are colored in wheat or green

Non-peptide small molecules, which usually noncovalently or reversibly interact with protein, are an important class of inhibitors against SARS-CoV M^pro^. These small molecules were discovered by a fragment-screening campaign or high-throughput screening or reasonable molecular design. Generally, compared with covalent inhibitors, these inhibitors have fewer side effects and lower toxicity. Recently, Jian and colleagues [131] gave a great description of non-peptide SARS-CoV M^pro^ inhibitors from 2010 to 2020, involving the structural characteristics, binding modes and SARs. However, difference in binding mode of between peptide and non-peptide inhibitors that plays a key role in discovery of new M^pro^ inhibitors were not illustrated in detail by them. Herein, we conduct a contrastive analysis of the superposition mode of co-crystal complex to understand this difference (Fig. 17). Akaji et al. [132] described that a novel decahydroisoquinolin scaffold in compound **33** contained an aldehyde group with prominent activity against SARS-CoV M^pro^ (IC_50_ of 108 μM). According to the superimposition of **N3** (a representative peptide inhibitor, Fig. 10b) and compound **33** bound to SARS-CoV M^pro^, the hydrophobic interaction in the large S2 pocket and the H bonds in the S1 pocket are consistent. The aldehyde group in **33** was expected to target residue Cys145 to form a stable C–S covalent bond, which is consistent with the Michael acceptor moiety in **N3**. The Ala-isoxazole moiety in N3 occupied the S4 pocket and forms a hydrogen bond interaction with residue Thr190, which is not observed between **33** and the S4 pocket. Jacobs et al. [133] reported the identification of a representative non-peptidic inhibitor **34** with excellent activity against SARS-CoV M^pro^ (IC_50_ of 4.8 μM) through virtual screening approaches. As shown in Fig. 17c, the superimposition of **N3** and **34** bound to SARS-CoV M^pro^ have confirmed that the 3-pyridyl group, the tert-butyl anilido group and tert-butyl amide group of **34** occupied the S1, S2 and S3 pockets, respectively, similar to the results obtained with the P1, P1′, P2 and P3 of peptide inhibitor **N3**. Compound **34** was a noncovalent SARS-CoV M^pro^ inhibitor different from most reported peptide inhibitors that act via covalent modification of the enzyme. Turlington et al. [134] reported the biological activity of a series of non-covalent benzotriazole derivatives from the NIH Molecular Library Probe Production Center Network (MLPCN). Compound **35** (Fig. 17a) was identified as the most potent anti-SARS-CoV M^pro^ inhibitor with an IC_50_ of 6.2 μM. As shown in Fig. 17d, for compound **35**, the key H bonds between the benzotriazole moiety and S1 pocket, the hydrophobic interaction between the 4-acetamidoaniline moiety and S2 pocket, and the two H bonds formed with residue Glu166 were observed, which was also observed for compound **N3**. Overall, peptide inhibitors aim to achieve irreversible inhibition effects by the formation of a C–S bond between electrophilic warhead groups and residue Cys145. Non-peptide small molecules usually utilize a noncovalent mechanism of action to accomplish efficient inhibition effects involving H bonds, hydrophobic interaction and van der Waals forces.

## Conclusion

The COVID-19 pandemic has dealt a painful blow to the safety of human life and the economic development of the world. A novel coronavirus, SARS-CoV-2, has been identified as the causative agent of the disease. Although extensive investigations are being carried out to fight SARS-CoV-2 infection, there are no specific antiviral agents available to manage this epidemic at present. Consequently, drug development against SARS-CoV-2 is an extremely urgent and challenging goal for which the understanding of the structural biology of SARS-CoV-2 and intimate knowledge of molecular structures involved in implementing infection and replication mechanisms are of crucial importance. It has been confirmed that the virus takes advantage of the host proteins TMPRSS2 and ACE2 to invade the human body, and RdRp and M^pro^ are vital in adjusting coronavirus replication and transcription of the viral life cycle. In this perspective, we mainly focused on the structural biology and medicinal chemistry of key proteins in the life cycle of SARS-CoV-2. Some of the successful and candidate inhibitors that have raised interest because of their effectiveness on homologous viruses in the coronavirus family are also summarized in this report. The protracted time needed for new drug development makes it highly desirable to repurpose existing antiviral agents that have been approved for clinical use against homologous viruses whenever they evidence effectiveness against SARS-CoV-2. Because many antiviral targets show highly conserved sequences among viruses in the coronavirus family, this underscores the reasonable possibility of rapid identification of both existing drugs and modifications to existing drugs that will enable effective control over the spread of COVID-19. Evaluation of these begins with an understanding of the modes of action of some representative broad-spectrum antiviral drugs, especially those against the homologous virus SARS-CoV. These are summarized in this report with the hope of driving the development of broad-spectrum inhibitors against SARS-CoV-2. In conclusion, we believe that this review will be useful to aid the research community in the discovery of novel and potent anti-SARS-CoV-2 inhibitors for distribution to clinics in the near future.

## Abbreviations

ACE2: Angiotensin-converting enzyme 2
COVID-19: Coronavirus disease 2019
CLD: Collectrin-like domain
EC_50_: Half-maximal effective concentration
IC_50_: Half-maximal inhibitory concentration
M^pro^: Main protease
NAA: Neutral amino acid
NTD: N-terminal domain
PL^pro^: Papain-like protease
PNCC: Partial negative charge cluster
RBD: Receptor-binding domain
RBM: Receptor-binding motif
RdRp: RNA-dependent RNA polymerase
RMSD: Root mean square difference
RTP: Remdesivir triphosphate
SARS-CoV-2: Severe acute respiratory syndrome coronavirus 2
TMPRSS2: Transmembrane protease serines 2
